# A study protocol outlining the development and evaluation of a training program for frontline managers on leading well-being and the psychosocial work environment in Danish hospital settings – a cluster randomized waitlist controlled trial

**DOI:** 10.1186/s12889-023-15728-2

**Published:** 2023-05-10

**Authors:** V. L. Dalgaard, A. Gayed, A. K. L. Hansen, R. Grytnes, K. Nielsen, T. Kirkegaard, L. Uldall, K. Ingerslev, J. Skakon, C. B. Jacobsen

**Affiliations:** 1 Department of Psychology and Behavioural Sciences, Aarhus University, Bartholins Allé 9, 8000 Aarhus C, Denmark; 2 Crown Prince Frederik Center for Public Leadership, Aarhus University, Bartholins Allé 7, 8000 Aarhus C, Denmark; 3 Black Dog Institute, University of New South Wales, Hospital Road, Randwick, NSW 2031 Australia; 4 Department of Occupational Medicine, Danish Ramazzini Centre, University Research Clinic, Regional Hospital Goedstrup, Moellegade 16, 7400 Herning, Denmark; 5 Sheffield University Management School, The University of Sheffield, Conduit Road, Sheffield, S10 1FL UK; 6 Central Denmark Region, Corporate Human Resource Development, Oluf Palmes Allé 32, 8200 Aarhus N, Denmark; 7 Department of Psychology, University of Copenhagen, Øster Farimagsgade 2a, 1353 Copenhagen K, Denmark

**Keywords:** Managers, Employees, Stress, Burnout, Job satisfaction, Hospital staff, Self-care, Staff-care, Leadership training, Health-oriented leadership

## Abstract

**Background:**

Hospital staff are often exposed to stressful psychosocial working conditions and report high levels of stress and burnout, which may negatively impact the safety of employees and patients. Managers hold unique knowledge of workplace conditions and needs of employees, but leadership interventions to improve the well-being of managers and employees in hospital settings are scarce. This study evaluates the effects of a leadership intervention based on a health-oriented leadership approach on the well-being and psychosocial work environment aspects of managers and employees.

**Methods/design:**

The study is designed as a randomized, waitlist-controlled trial with two groups (intervention and waitlist control group) and measurements at baseline, 6- and 12-month follow-up. We aim to include 200 frontline managers in Danish hospital settings and their approximately 5,000 employees. The leadership training comprises five full day modules and four smaller group-training sessions over a period of 5 months. The main aim is to improve stress, burnout, self-care, and perceived level of staff-care among managers and employees. Sickness absence will also be assessed at both manager and employee level. In addition, several psychosocial factors will be assessed at the employee level. A quantitative and qualitative process evaluation will also be conducted.

**Discussion:**

Action towards supporting the mental health of hospital employees is important to maintain a strong healthcare system. There is increasing recognition that best practice in workplace mental health requires an integrated approach that prevents harm and promotes positive mental health. There is also increasing understanding of the key role managers play in maintaining well-being within the workplace, however they often report a lack of knowledge and skills to promote employee mental health. The current leadership training program has been developed for frontline managers working in a hospital setting. The aim is to increase managers’ application of strategies to facilitate a healthy psychosocial work environment to benefit well-being and mental health among staff and managers themselves.

**Trial Registration:**

The study was retrospectively registered on November 21, 2022 in Clinical Trial.gov with identifier: NCT05623371.

**Supplementary Information:**

The online version contains supplementary material available at 10.1186/s12889-023-15728-2.

## Background

Healthcare employees often report high rates of stress, depression, and burnout [1, 2]. These conditions have high personal consequences on quality of life and work trajectory, and are associated with reduced patient safety outcomes [3], medical errors [3, 4], and lower quality of patient care [5]. Work-related risk factors such as high job-demands, low job control, long working hours, organizational injustice, and low degree of social support have all been identified as potential sources of stress and mental health problems in the workplace [6, 7]. These mental health risk factors often characterize the working conditions in hospital settings [8, 9]. In a comparison to 38 other occupational groups in Denmark, Danish hospital workers are among the seven most exposed groups to risk factors such as low influence, low leadership quality and support, low recognition and cooperation and high job demands and work tempo. Hospital workers were also among the groups with the highest levels of perceived stress and the lowest levels of job satisfaction [10].

Retaining qualified healthcare employees is a challenge in countries across Europe [11, 12] and several studies have demonstrated that adverse work environmental factors are associated with higher turnover in hospital staff. A recent study estimated that up to 44% of staff turnover among Danish hospital employees could potentially be prevented by improving the psychosocial work environment [11]. The psychosocial work environment may be understood as a concept that involves job and work environment aspects such as organizational climate or culture, work roles, interpersonal relationships at work, and the design and content of tasks (such as variety, repetition, meaning) [13].

Managers hold unique knowledge of workplace conditions and of the needs of employees they supervise. They can play an important role in the design of a healthy psychosocial work environment through their authority, by adjusting working conditions and modifying existing practices and work processes to minimize the potential harm to staff from work-related mental health risk factors [14, 15]. Managers also engage directly with employees and are in the position to act as role models in the workplace [16, 17]. Thus, a number of studies have identified associations between supportive leadership behavior and positive mental health outcomes for staff members [6, 18–21] including reduced risk of sick leave [22]. A recent review by Løkke [23] found that leadership behaviors, attitudes and social modelling had an influence on employee absence with manager behavior playing an essential role in determining occupational outcomes of stressed and absent employees.

Despite the potential to positively impact mental health outcomes of employees, studies suggest that managers often feel hesitant or lack sufficient skills to address mental health issues among staff they supervise [21, 24, 25]. Manager confidence in managing well-being and mental health within the workplace has been found to be a key predictor of their engagement to initiate conversations about mental health matters with staff [21]. With the aim of increasing implementation of supportive manager behaviour, more studies are required that develop and test models for how managers in hospitals can be trained in skills that increase their confidence and likelihood to engage in such behaviours.

To do so, this study protocol outlines a proposed randomized waitlist controlled trial designed to evaluate an evidence-based leadership training intervention focused on strengthening leadership skills pertaining to the psychosocial work environment and the well-being of employees and the managers themselves. We aim to collect data from 200 frontline managers and 5,000 employees within a Danish hospital setting to evaluate the potential impact on both manager- and employee mental health outcomes of leadership training delivered to the healthcare managers.

The well-being and working environment of managers is often overlooked. Studies show that although managers overall value ​​the leadership duties related to their role, some factors can be experienced as burdensome [19]. One such factor may concern the experience of cross pressure, where a person socially or psychologically is affected in opposite directions [26]. For example, in terms of work coherence, the middle manager makes an effort to foster top management interests and implement strategic decisions, while at the same time the employees' interests and well-being must be looked after in the best possible way, although demands sometimes collide. Other stress-related factors for the manager includes imbalance between demands and resources, time pressure and experience of loneliness associated with lack of support from top management and competition within the management group [19, 27]. The experience of loneliness is supported by a study from Ladegaard et al. [28], where middle managers reported an experience of feeling alone, although they struggle with the same challenges as their managerial colleagues. Therefore, in addition to implementing strategies to support employee mental health, managers may also need to address their own well-being, as a means to leading by example and creating a team culture in which their employees can thrive [29, 30].

Leadership training studies predominantly investigate relatively generic content such as team-building, producing results, and influencing others [31], whereas studies evaluating on training which focuses on issues related to the workplace mental health of employees are less described and investigated, particularly in hospital settings [8, 18]. Recent studies have attempted to evaluate the concept of health-oriented leadership as a framework for designing leadership interventions and understanding leadership behaviors aimed at improving well-being and reducing symptoms of stress and burnout in managers and their employees [16, 29]. Health-oriented leadership may be understood as a general behavioral and organizational preventive approach to health, comprising aspects focused on managers as well as staff. Manager aspects involve mindsets, attitudes/beliefs and behaviors of managers that are potentially influential of the managers own health-related behavior and perceptions of well-being/stress [8]. This approach takes into account the well-being of the manager and suggests that the manager’s stress level and self-care is an important antecedent of staff members’ stress and self-care through a potential spillover effect of the role model function of managers [29], and through the influence of manager well-being on the managers’ behavior and contact with staff [8]. Thus, the behaviors of strained managers have been known to influence the well-being of staff members negatively. Staff aspects of health-oriented leadership involve the managers’ values, awareness, and behavior towards their employees’ health and well-being [19, 20]. Values refer to interest and attached importance of employee health, behavior refers to the managers’ personal activity and engagement in health-oriented actions such as reducing risk factors and creating a healthy work environment. Awareness refers to attention, sensitivity, and reflection [25]. Manager self-care and staff-care are expectedly related in the sense that managers, who are able to take care of their own health are in a better position to take care of their staff’s health and support and develop staff towards self-care.

A recently published systematic review examined the impact of health-oriented leadership training studies in the healthcare sector [8]. Seven studies were eligible according to inclusion criteria. Overall, results suggested that interventions with reflective and interactive elements in a group-based setting seem to be the most effective in improving mental health outcomes among employees. However, only four of these studies showed a positive impact on either manager or staff health [8]. In summary, leadership interventions aimed at improving employee well-being in healthcare settings seem to have been almost non-existent and as stated by Stuber et al. [32], hardly any information is available on change potential, feasibility and acceptance of these interventions among hospital managers and staff.

Looking beyond the healthcare specific context, other reviews have been conducted on studies of manager training and its impact on employee stress/well-being outcomes. A Cochrane review by Kuehnl et al. [33] examined the impact on employee stress, well-being and absenteeism of the implementation of HR training of managers. The training content of included studies was specifically designed for managers and aimed at improving the manager-employee interaction and managers’ ability to design the work environment. The review rendered evidence of any positive effect of leadership training on employee outcomes, inconclusive. Overall, Kuehnl et al. [33] concluded that the quality of existing studies is very low. Similar results were found in a meta-analysis of leadership mental health training conducted by Gayed et al. [34] due to the limited number of studies with employee level outcomes available. This meta-analysis recommended a need to investigate the effects of leadership training on the mental health and well-being of the employees further. However, a few recent randomized controlled trials on the effect of skills-based leadership training on staff mental health have found that training improves manager confidence to support employee mental health matters, and increase their self-reported application of recommended managerial practices in workplace mental health towards their employees [34–36]. Other positive impacts from such interventions include improvements in managers’ knowledge of mental health, with one study also reporting a reduction in sickness absence among employees [35].

A major challenge in leadership training relates to *transfer of training* in terms of how the skills learned during training are translated into subsequent changes in leadership behavior and maintained over time [37]. Factors such as trainee characteristics, training design and various organizational conditions (including employees’ and managers’ perceptions of the intervention) may directly hinder or improve training transfer [38]. To understand the mechanisms behind any intervention effect, such conditions should be tracked by a process evaluation conducted throughout the study [39–41]. The leadership training literature recommends learning principles such as experiential learning and action learning to increase transfer. These principles can provide managers with time and opportunities for reflection and practical experience with new approaches and behaviors and improve their ability to transfer meta-skills to changing demands and conditions in actual work situations [31].

In summary, there is a need for more rigorous intervention-based research with a focus on how health-promoting leadership training can effectively transfer to make a difference for the well-being and work environment of managers and their employees.

With this in mind, a training program co-designed by the research group and organizational human resource ( HR) consultants of the recipient organization was developed based on the health-oriented leadership approach and with the intention of fitting the training to the organization as suggested by guidelines for organizational interventions [42]. The training program was designed to increase managers' skills in addressing their own and employee well-being as well as supporting a healthy psychosocial working environment, i.e. reducing risk factors, promoting protective factors and a supportive work culture [2, 29].

More specifically, this study aims to:

- 1) Determine whether a comprehensive leadership training program can positively impact the perceived self-care and staff-care behavior of managers and the self- and experienced staff-care of employees as well as the stress, burnout and well-being levels of both managers and employees.
- 2) Determine whether this same leadership training program can positively impact sickness absence and intention to leave among managers and employees.
- 3) Determine whether this same leadership training program can improve the psychosocial working environment of employees.
- 4) Identify potential organizational facilitators and barriers for conducting the training as planned.
- 5) Investigate organizational facilitators and barriers for transfer of training elements in the daily practice of managers subsequent to undertaking the training program.

The training program is described in more detail below.

## Methods

### Study design and participants

**Fig. 1:**
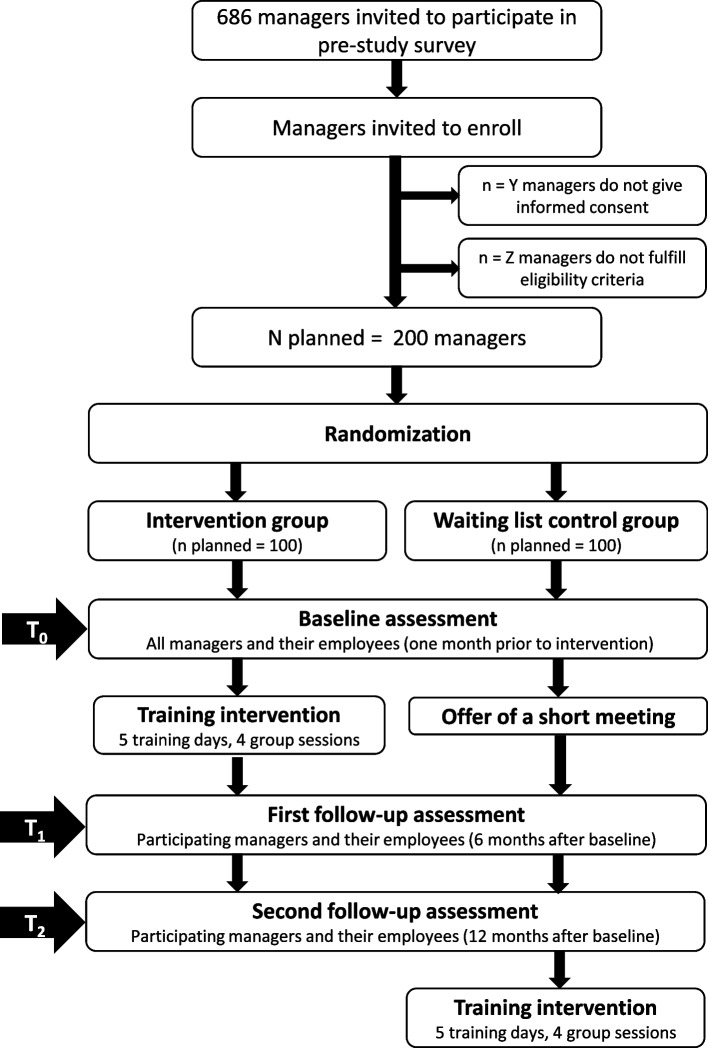
Flowchart of the study design

The project will investigate the effects of training frontline managers in hospitals in the Central Denmark Region through a cluster randomized waitlist controlled trial. The project aims to include 200 managers who, after registration and informed consent for the research project, are randomly allocated to receive either leadership training commencing in the beginning of 2023 (intervention group), or leadership training commencing in the beginning of 2024 (waitlist control group). Recruitment began in May 2022 and ended during January 2023. The participating managers are randomized by hospital ward, but the vast majority of wards include only one participating manager. Because some managers are clustered in wards, the wards are stratified into three groups of wards with: 1. One participating manager in the ward, 2. two participating managers, and 3. three or four participating managers. In each strata, wards are randomly assigned to equal sized intervention and control groups. For each strata, we randomly number all wards and using the RAND function in Excel, each ward is assigned with a random number between 0 and 1. The lowest 50% of numbers are assigned to the intervention group and the other 50% are assigned to the control group. In addition, the intervention group is randomly assigned to five training groups. The randomization is conducted by a neutral research assistant. All managers and their employees will be invited to complete outcome questionnaires at baseline prior to training, as well as 6-months and 12-months post baseline. An overview is presented in the flowchart in Fig. 1. Managers will also receive a short questionnaire related to the process evaluation following completion of each training module to assess the learning experience (see further description of the process evaluation below).

### Inclusion and exclusion criteria

To be eligible to participate, individuals must currently be employed as frontline managers with staff responsibility within a Central Denmark Region hospital. Frontline managers include department nurses, senior therapists, senior medical laboratory technologists, senior medical secretaries and service- or production leaders. Clinical directors are excluded by default, as they have departmental responsibility and no direct line manager responsibility. Similarly, chief physicians are excluded if they only have functional responsibility and no staff responsibility. Participation is voluntary and recruitment occurs through notices, information sent to main department mailboxes and direct contact with hospitals and the corporate HR Management Forum for top management of the respective regional hospitals. All eligible managers can sign up to participate through the regional online platform for hospitals. If a manager does not meet the above criteria, they are not offered a place in the leadership training study. It is also essential that participants can speak and understand Danish, as the training modules, questionnaires and participant information is provided in Danish. Managers are required to provide informed consent to participate in the project. This takes place by ensuring that the managers have access to extensive information about the project before they sign up.

### Identification of employees for quantitative assessments

As we aim to assess the effect of a leadership training intervention on the well-being and psychosocial work environment of the employees of the participating managers, we include all the employees for whom the participating managers have direct staff responsibility. In this study, direct staff responsibility is understood as being responsible for conducting annual employee assessments or similar, and thereby being responsible for the well-being and the psychosocial environment of the employee in the workplace. We identify the employees through HR-data on formal manager-employee hierarchies. To account for informal management structures, which may disrupt the clarity of manager-employee links, each participating manager is asked to validate a list of employees provided by the researchers. To account for turnover, this task is to be completed before each questionnaire follow-up.

### The intervention

The leadership training consists of five full-day training modules over five months from the beginning of the training delivered to groups of up to 20 participants. There will be additional meetings in smaller practice groups between each module to solidify content learned in the training modules.

The training modules are facilitated by two experienced Human Resource Development (HRD) consultants, employed in Corporate HRD in the Central Denmark Region and with expertise in organizational psychology and leadership development within the region. Short video presentations of research-based knowledge will be provided during the modules by researchers with expert knowledge in subjects relevant for the training.

The training group meetings between the modules will focus on transferring the learning from each module into the managers' daily working context. The first of the meetings will be facilitated by an HRD consultant to support a meeting structure and the psychological safety in the group and the training. The next three meetings will be conducted by the group itself.

During the waiting period, the control group will have the opportunity to participate in a meeting with introductory information on the project and the psychosocial work environment.

**Fig. 2:**
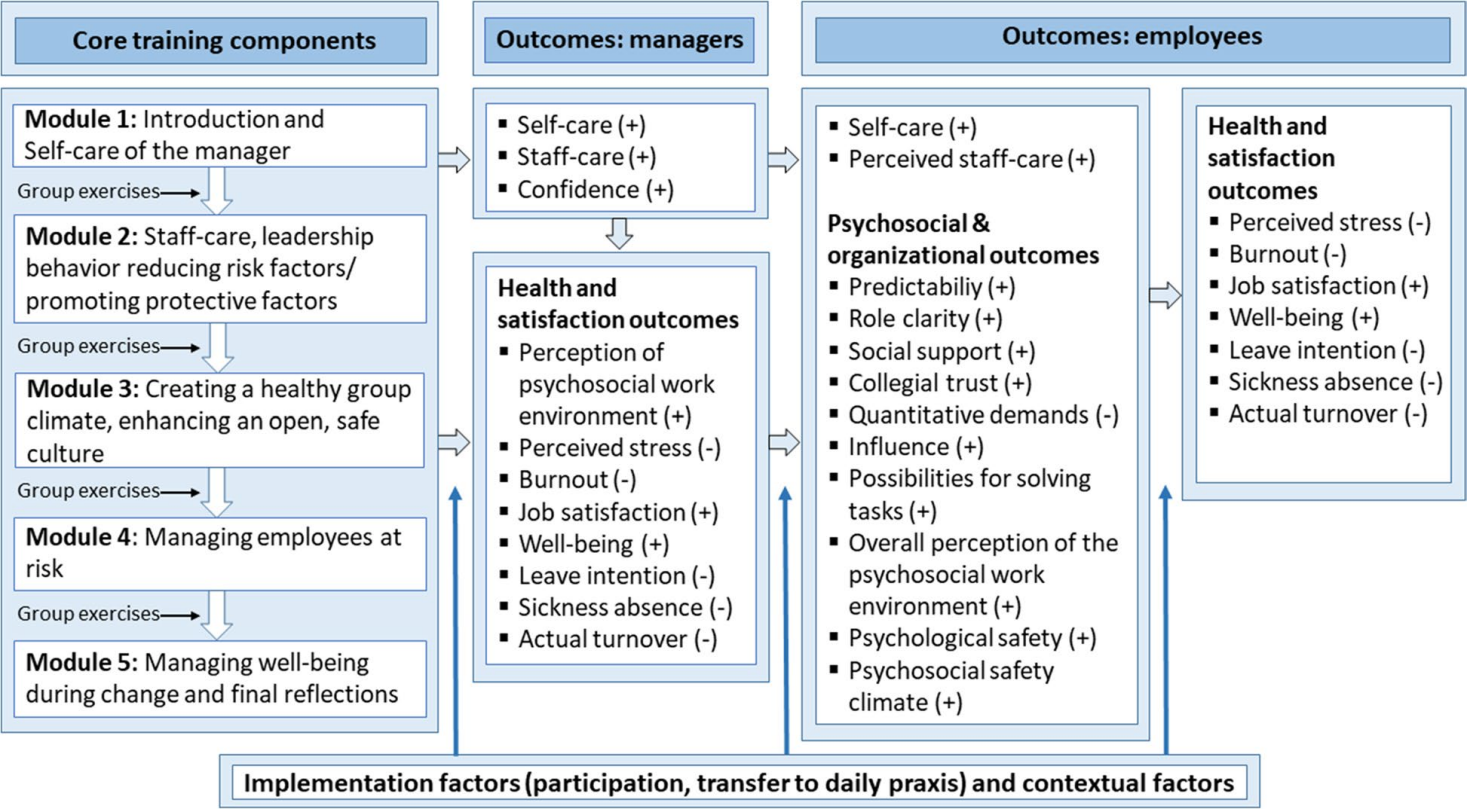
Program theory of the study

The leadership training is based on existing research, but to ensure a good fit between the provided training and the target group, we incorporated information obtained through a needs assessment survey conducted in 2022. The survey focused on the working environment, stress, well-being and needs of managers when addressing employee well-being. Insights from a pilot test conducted in autumn 2021 were also included in the design of the training. The pilot test consisted of 14 managers from the Region being trained during 6 months in psychosocial subjects regarding self-care and staff-care. The training program described in the current study was co-developed by the researchers and the HRD consultants who facilitated the pilot and will participate in the training of this study. The training incorporates evidence-informed content examining the benefits of health-oriented leadership and inspired by existing studies within this or similar frameworks. Thus, the training aims to address both the well-being of the managers (self-care) and behavior toward the well-being of their employees (staff-care). With regard to the former, the training will include subjects such as knowledge of stressors, self-care in terms of stress management such as mindfulness exercises, recovery and coping with cross pressures in the leadership role and reflections behind the motivation of being a manager. For the latter, the training will cover topics such as leadership behavior, communication, handling conflicts, reducing risk factors and promoting protective factors in the psychosocial working environment, creating a safe work culture, addressing employees at risk of stress and mental health problems, overload reactions, return to work and employee well-being during change. For an overview of the modules, please see Fig. 2 below. The content of the intervention was initiated by the researchers based on scientific literature and co-developed with the HRD consultants responsible for facilitating the training. All training modules will end with participating managers working on individual action plans to transfer learned skills to their daily work lives. Each module will contain elements of knowledge, reflections and action/behavior to bring both the value, awareness, and behavior dimensions from health-oriented leadership in play and thereby enhance the likelihood of training transfer.

### Program theory

The program theory and the outcomes intended to change by the intervention are depicted in Fig. 2. The left side of Fig. 2 provides an overview of the core training components and the structure of the training. The training content is intended to promote self-care, staff-care and confidence in managers, which in turn should improve health and satisfaction outcomes in managers. Changes in manager self-care, staff-care and confidence may also lead to both increased self-care and perceived staff-care among employees. Increased staff-care among managers should also lead to improvements in psychosocial protective factors and team culture regarding psychological safety and elements of the psychosocial safety climate. Changes in employee self-care, perceived staff-care and improved psychosocial outcomes should improve the distal intervention outcomes such as employee mental health and satisfaction outcomes including mental health measures, sickness absence and retention. The expected change in outcomes over time (increase or decrease) is illustrated by ± .

### Primary and secondary outcomes

The registered primary outcomes at clinical trials.gov for both managers and employees are perceived self-care and staff-care (see Fig. 2) as well as perceived stress, burnout, job satisfaction and sickness absence.

The registered secondary outcomes for both managers and employees are well-being, the overall perception of the psychosocial work environment as well as turnover intention and, actual turnover.

Additional registered secondary outcomes for only managers was confidence in addressing mental health issues and the psychosocial work environment. For only employees additional secondary outcomes were central aspects of the psychosocial work environment (predictability, role clarity, influence, social support from colleagues and managers, leadership quality, trust between colleagues, demands, and possibilities for solving work tasks), psychological safety and the psychosocial safety climate. Table 1 displays the SPIRIT flow diagram for the schedule of enrollment, intervention and assessment. Specification of measures and data collection is provided below.

**Table 1:**
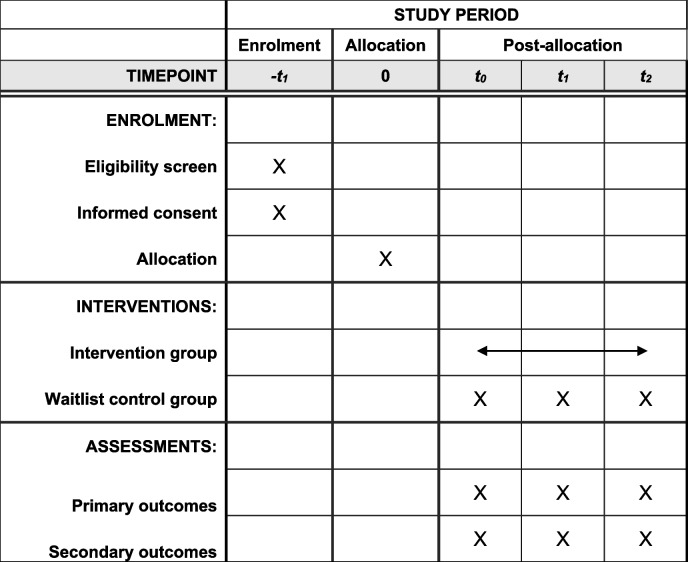
SPIRIT flow diagram of the schedule of enrollment, intervention and assessment of the study

### Data collection (effect evaluation)

The participating managers randomly allocated to the intervention and waitlist control group and all their employees, will be invited to complete questionnaires at baseline, 6 months follow-up and 6-months post training (12 months follow-up). Register data on sickness absence and staff turnover will be retrieved for the one year period prior to and following baseline for all managers and employees. In addition, a process evaluation will be conducted including both quantitative and qualitative data (see description below).

#### Quantitative assessment

The quantitative assessment encompasses both questionnaire data and register data on participating managers and their employees.

#### Questionnaires

The three questionnaires are sent out to the participating managers and their employees at three points in time (baseline, 6 months follow-up and 12-months follow-up).

#### Single items

##### Sociodemographic data

The participating managers and their employees will be asked to provide their gender, age, marital status, children living in the household, and highest educational level attained.

##### Work-related data

Regarding their current employment, we ask the managers about their managerial experience (years), their average number of working hours, and whether they have received leadership training prior to signing up for this program. Through HR-data, we were able to assess span of control (the number of employees for whom each manager is responsible), job titles, and which professions their employees represent. Furthermore, the managers are asked about their general job satisfaction [43] and turnover intention. The employees were asked about their average working hours, job satisfaction, the overall perceived psychosocial work environment [43], and their turnover intention. We also asked employees to assess a question we developed on the perceived quality of their care when in contact with patients.

##### Health-related data

Both participating managers and their employees were asked to self-report their health status, whether they have been on sick leave due to stress or mental illness during the past 12 months, whether they had been prescribed medication for anxiety or depression, sleeping pills or tranquillizers, and to report on their quality of sleep.

#### Scales

##### Health-oriented leadership

Health-oriented leadership [29] focuses on leadership targeted towards improvement of physical and psychosocial health and has been measured and validated by Franke, Felfe and Pundt [29]. In this study, we use a version adapted to target well-being and mental health, which predominantly focuses on factors relevant for the psychosocial work environment. Health-oriented leadership comprises two dimensions: Self-care (16 items) and staff-care (18 items). A sample item appearing in both scales is “I notice in time, when I need a break”/ “I notice in time, when my employees need a break.” The items are answered on a five-point Likert-scale from “To a very low degree” (1) to “To a very large degree” (5). Self-care and staff-care scales will be administered to both managers and employees.

##### Perceived confidence

This scale measures the extent to which the participating managers feel confident in handling different aspects of the psychosocial work environment [18]. The managers will be asked to state their current level of confidence with the task. A sample item is “Initiating contact with staff on sickness absence leave that you believe might be due to mental illness”. The items are answered on a five-point Likert scale from “Not at all confident” (1) to “Extremely confident (5).

##### Cohen’s Perceived Stress Scale (PSS-10)

The PSS-10 is a 10-item scale [44] measuring the extent to which the participating managers and their employees feel that their life is stressful, unpredictable and uncontrollable on a scale from 0–40. A sample item is “In the last month, how often have you been upset because of something that happened unexpectedly?” Each item is answered on a five-point Likert-scale ranging from “Never” (0) to “Very often” (4).

##### Copenhagen burnout inventory

The Copenhagen Burnout Inventory [27] measures both personal, work-related, and client-related burnout. In this study, we only include the measure of personal burnout – a state of prolonged psychical and psychological exhaustion. The scale goes from 0–20. A sample item is “How often do you feel tired?” Each item is answered on a five-point Likert scale ranging from “Never/almost never” (0) to “Always” (4).

##### WHO well-being (WHO-5)

WHO-5 is a five-item measure [45], uncovering general well-being, by asking respondents to indicate how they have felt for the past two weeks. A sample item is “Over the last few weeks I have felt cheerful and in good spirits”. The items are answered on a six-point Likert-scale ranging from “At no time” (0) to “All the time” (5).

##### Danish Psychosocial Working Environment Questionnaire (DPQ)

The DPQ is a comprehensive tool, focusing on a variety of factors in the psychosocial work environment. The full DPQ consists of 119 items covering 38 different psychosocial work environment dimensions. We include some of the questions, specifically questions on predictability, role clarity, influence, social support from colleagues and managers, leadership quality, trust between colleagues, demands, and possibilities for solving work tasks. The items are answered on a five-point Likert-scale ranging from “Always” (0) to “Never” (5).

##### Psychological safety

We ask both the participating managers and their employees to answer a five-item scale on psychological safety [46], measuring the degree to which people in their workplace feel safe in taking the risk that comes with speaking up and sharing concerns. A sample item is “If you make a mistake in this unit, it is often held against you.” The items are answered on a five-point Likert scale ranging from “Completely disagree” (1) to “Completely agree” (5).

##### Psychosocial Safety Climate (PSC-12)

We adapted six-items from the PSC-12. The PSC-12 measures the extent to which the management protects the mental health and safety of their employees. A sample item is “Management clearly considers the psychological health of employees to be of great importance”. The items are answered on a five-point Likert scale ranging from “Completely disagree” (1) to “Completely agree” (5).

##### Symptom Checklist (SCL-90)

The SCL-90 [47] can help determine the existence psychological symptoms. Nine dimensions can be evaluated. We included the dimensions for depression and anxiety. Participating managers and employees are asked to what extent symptoms has bothered or stressed them during the last week. A sample item is “Nervousness or shakiness inside”. Each items is answered on a five-point Likert scale ranging from “Not at all” (0) to “Extremely” (4).

#### Register data

By utilizing access to social security numbers, we will follow the participating managers and their employees in HR-registers and Statistics Denmark, to unfold how the training affects sickness absence and turnover. These data will be collected starting from January 2022 to January 2024.

Sickness absence outcomes at both manager and employee level will comprise:

- 1) The percentage of sickness absence six months prior to baseline, from baseline to first follow-up and from first follow-up to second follow-up.
- 2) The percentage of sickness absence 12 month prior to baseline and from baseline to second follow-up.

Turnover outcomes at the manager level will comprise:

- 1) Number of managers leaving in each group

Turnover outcomes in relation to employees will comprise:

- 1) For the time periods from six months prior to baseline, from baseline to first follow-up and from first follow-up to second follow-up turnover percentage is estimated as the number of employees, who have left their job, divided by the span of control of the manager.
- 2) Turnover percentage estimated as the number of employees, who have left their job, divided by the span of control of the manager for the time period 12 months before to baseline and from baseline to 12 months after.

#### Collecting the data

The questionnaire data is collected via the online Qualtrics software (Qualtrics, Provo, UT). At each time point, participating managers and their employees will have at least three weeks to complete the questionnaire. During these three weeks, three reminders will be sent out to those who have not yet completed the questionnaire. Register data will be collected retrospectively once the 12 months follow-up period is complete.

#### Sample size

We estimated the number of participants using a power calculation based on survey measures and an expected moderate effect size of 0.54 (consistent with previous studies). We have adjusted for an individual baseline correlation of 0.5 and a group size of 20 with an expected intra-group correlation of 0.02. Participation is voluntary throughout the project and we expect a dropout rate of up to 20%. Based on these assumptions, we anticipate being able to recruit 100 managers per group and successfully to follow up on 80 per group. This would result in a satisfactory power of 0.83 using an alpha value of 0.05.

#### Statistical analyses

To evaluate the intervention effects, we will examine group differences in the measured outcome variables over time. The analyses will consider the clustering of observations of staff within the team of the manager, as well as correlation of the repeated measurements within each staff member. The effect of the intervention will be estimated by three quantities: The difference between groups in mean change from T0 (baseline) to T1 (6-months), T0 to T2 (12-months) and T1 to T2 in the intervention groups versus the control group. Analyses will be conducted as intention to treat and where relevant compared to per protocol analyses. In general, the estimation will be based on a mixed model with time, group and an interaction of group and time as fixed effects. For outcomes measured at manager levels, the mixed model will include a random level for training group, hospital ward and manager. For outcomes measured at the employee level, an additional random level for employee is included. Analyses at the employee level will be further adjusted for employee age, gender, occupation, and span of control of the manager. Possible bias due to missing data will be investigated with a set of sensitivity analyses. Regarding turnover among the managers during the follow-up period, the two groups will be compared by binomial regression corrected for occupation, age, gender. Models will be bootstrapped over ward. The data analyst will be blinded as to who is in the intervention or control group.

### Data collection (process evaluation)

The process evaluation will be based on realist evaluation [48, 49]. The key question that a realist evaluation seeks to answer is: What works for whom under which circumstances? The questions are answered through the test of context, mechanisms and outcome (CMO) configurations, i.e., exploring in which Contexts an interventions Mechanisms are triggered, to bring about the intended Outcomes [48]. Although realist evaluation has received limited attention in the training literature, recent frameworks have argued that realist evaluation may be used to understand the impact of training interventions to improve employee mental health integrating a training transfer perspective [50, 51]. For example, it has been found that important mechanisms for learning and intention to transfer (intermediate outcomes) are the design and format of the training, as well as participants’ satisfaction with the training [38]. Key contextual factors that may influence how participants react to training are factors such as readiness for change, support from senior management and the extent to which participating managers feel the training fits in their daily work context [50]. Thus, contextual factors involve both the organizational context and individual characteristics [50]. Our outcomes are listed below.

Nielsen and Shepherd [50] suggested a chain of effects, with intermediate outcomes: The extent to which participants feel they have acquired new learning (first proximal outcome) and intend to transfer this learning (second proximal outcome) will result in transfer attempts to translate the acquired learning (third proximal outcome) into new leadership behaviors (fourth proximal outcome), which potentially should lead to improvements in employees’ mental health and wellbeing (distal outcomes). At each identified step, contextual factors may influence the extent to which the outcomes are brought about by the mechanisms intended to facilitate the outcome and these will be captured by both the quantitative and qualitative process evaluation.

The current process evaluation was inspired by the Integrated Training Transfer and Effectiveness Model (ITTEM) framework developed by Nielsen and Shepherd [50] that integrates training transfer and training effectiveness. The ITTEM framework suggests that pre-and post-training contextual factors at both the individual and organizational levels influence the extent to which training transfer mechanisms are triggered. Thus, an advantage of this model is that it links the concept of transfer to outcomes and training effectiveness (i.e., whether training is actually transferred to and integrated in new practices at the workplace as well as if the relevant training outcomes are achieved).

The process evaluation will use both quantitative and qualitative data to test the CMOs of the study, which are outlined below:

• If managers are highly motivated for training and have volunteered to participate in training, then they are more likely to participate in all training modules and as a result, managers will report learning as an outcome of the training (proximal outcome for managers).
• If managers are confident about managing the psychosocial work environment, then they will engage in more transfer attempts and as a result they will be likely to report improvements in staff-care (intermediate outcome) with reduced stress and burnout and increased job satisfaction in employees as a result (distal outcomes for employees)
• If managers experience senior management support, then they will engage in more self-care activities (intermediate outcome) and as a result, managers will report improvements in wellbeing, stress, and burnout (distal outcomes for managers)
• If managers experience supportive senior managers, they will engage in more transfer attempts and as a result they will report larger improvements in staff-care (intermediate outcome), which will result in lower levels of stress and burnout and increased job satisfaction among employees (distal outcomes).
• Managers, who experience the training as relevant and addressing the challenges in their everyday work, will have more transfer attempts, and as a result they will increase staff-care (intermediate outcome), which will result in larger improvements in psychosocial work environment outcomes (intermediate outcome) leading to improvements in health and satisfaction outcomes of employees (distal outcomes).
• Managers, who experience a higher degree of intention to transfer, will have not only more, but also more diversified and repeated transfer attempts (proximal outcome), and as a result more both self-care and staff-care behaviors (proximal outcome) leading to employees reporting higher levels of both self-care and perceived staff-care (proximal outcomes in employees).
• Managers, who experience staff shortage, will have fewer opportunities for transfer due to work pressure, and thus less transfer attempts, and as a result they will likely not increase their staff-care behavior (intermediate outcome) leading to a lack of improvement in stress, burnout and job satisfaction among employees (distal outcome in employees).

The purpose of these CMO’s is to obtain in-depth knowledge on what works for whom (e.g. how support from senior management is experienced or how they formulate their own needs in their daily practice) under which circumstances.

#### Quantitative process evaluation

A quantitative process evaluation will be conducted throughout the study during assessments at baseline, 1^st^ follow-up and 2^nd^ follow-up. In addition, all participating managers in the intervention group will be asked to answer a short survey following each module to assess their experience of the training.

Contextual factors that will be addressed are turnover in the hospital wards, span of control, and the support of senior management and subordinates of initiatives related to well-being and the psychosocial work environment. Other factors are time, resources, and opportunities to practice what is learned during the training and in the daily work setting of the managers. Cognitive, emotional, and behavioral aspects known to be influential of transfer will also be addressed such as perceptions of how the training and content fits the daily work of managers and various transfer elements such as confidence, intention, and motivation to transfer, acceptability of the training and experience of integrity of the training will also be assessed. Please see specifications below.

##### Quantitative process measures

To estimate the central aspects that relate to training transfer, selected items from the Generalized learning transfer system inventory will be employed [52]:

Readiness for change will be assessed with: *“Before the training I had a good understanding of how it would fit my job-related development”.* The item is answered on a five-point Likert scale ranging from strongly disagree (1) to strongly agree (5).

Motivation to transfer will be assessed with: *“I get excited when I think about trying to use my new learning on my job.* The item is answered on a five-point Likert scale ranging from strongly disagree (1) to strongly agree (5).

Time pressure as a contextual factor is assessed with: *“My workload allows me time to try the new things I have learned”.* The item is answered on a five-point Likert scale ranging from strongly disagree (1) to strongly agree (5).

To assess whether new knowledge was acquired after each module the following item from the Questionnaire for Professional Training Evaluation [53] will be used after each module: *“ I know substantially more about the training contents than before”.* The item is answered on a five-point Likert scale ranging from strongly disagree (1) to strongly agree (5).

Fit of training content to the job will be assessed with three items: *“The activities and exercises the consultant(s) used helped me know how to apply my learning on the job”*, *“It is clear to me that the people conducting the training understand how I will use what I learn”, and “ The way the consultant(s) taught the material made me feel more confident I could apply it”.* All Items are answered on a five-point Likert scale ranging from strongly disagree (1) to strongly agree (5).

Intention to transfer [54] will be assessed after each module with the items: “*I believe what I learned on the training can help me at work*”, “*The skills I developed during the training will help me at work”, “I developed new skills for my work that I didn't have before*”. Both items are answered on a five-point Likert scale ranging from strongly disagree (1) to strongly agree (5).

To assess whether the participants experience sufficient opportunities to apply learned knowledge and skills, the following items by Holton [55] will be used after module 2 to 5 as well as in the 1^st^ and 2^nd^ follow-up: “*I have had the tasks necessary to apply the skills and knowledge I learned on the training*”,”*I have the necessary resources to use what I learned in training*”, and “*I have the information necessary to apply the skills and knowledge I learned on the training course*”. All Items are answered on a five-point Likert scale ranging from strongly disagree (1) to strongly agree (5).

To assess whether transfer was successful the following item from the Questionnaire for Professional Training Evaluation [53] will be applied during 1^st^ and 2^nd^ follow-up: “*In my everyday work, I often use the knowledge I gained in the training*”, “*I successfully manage to apply the training content in my everyday work*”, "*I am able to transfer the skills learned in training courses back to my actual job*”. Items are rated on an 11-point scale ranging from 0 (Therefore, an 11-point response scale ranging from 0 percent (completely disagree) to 100 percent (completely agree).

To assess the integrity and climate of the training, we will also ask the participants the following questions (also used by Vuori et al. [56]) during first follow-up: “*Did the consultant(s) make you feel like your participation was valued?*”, “*Did the consultant(s) do or say something that makes you think they understand your situation?*”, “*Did the consultant(s) encourage you to participate in the assignments they gave*”, “*Did you find the atmosphere friendly and encouraging*?”, “*Did you find group discussions useful?*”, “*Did you share your own experiences in the group*?”.

Support from senior management and colleagues will be measured with items already mentioned among the outcome measures previously described in this paper.

##### Analyses related to the process evaluation

Analyses of quantitative process data will be conducted through descriptive statistics and methods of investigating predictors of outcome change for example by adding process variables to quantitative models to investigate whether the variable was related to specific outcomes. The results of the effect evaluation, and the quantitative and qualitative process evaluation will be integrated to gain the highest level of insight into answering CMO’s listed above.

#### Qualitative process evaluation

Barriers and facilitators of adherence and transfer of training to daily practices will be explored through semi-structured interviews with the managers receiving training, their employees and other stakeholders such as the clinical directors. Participant observation of the training modules will also be conducted.

##### Interview data

Interviews will be conducted in order to gain in-depth understanding of the different parts of the leadership training and between module exercises [57], including how they influence each other as well as how the training is transferred to the daily practices and setting of the managers. The interviews will be conducted at different time points during the 5 months of the training and throughout the follow-up period. Individual interviews will be conducted with 14 randomly selected managers after Module 3 has been completed. Focus group interviews will be conducted with in total 10–15 participating managers at the end of Module 5. In addition, selected managers from each group will be interviewed five to six months after completion of the leadership training. Interviews are also conducted with 1–2 managers focusing on the experiences with group exercises, both between Modules 2 and 3, and between Modules 4 and 5. In addition, focus group interviews are conducted with 3–4 employees from 3 selected departments (from which managers participate in the training modules) after the training. The employee interviews are conducted as focus groups, as this interview format allows participants to discuss and nuance each other's views [58].

A semi-structured interview guide is prepared, i.e. the questions are developed and expanded as the modules and group exercises are completed. In this way, the benefits and possible challenges for the managers can be continuously identified and used to further explore and develop the configurations of the CMOs. The focus will be on the participants' own perspectives, partly on their own challenges in dealing with the psychosocial work environment, partly on their own well-being and health, and partly on their perception of what the context means for their challenges and their attempts to transfer their learning from the training. Examples of questions from the semi-structured interview guide could be: Can you describe your own motivation or reasons for signing up to the Matterhorn-training program? How do you believe that the training program can help you to improve your psychosocial management in the department? How does the form of the training, and the different formats, fit your own preferred way of learning – can you use it, in daily practice? How, in your view, has the employees benefitted from your participation in the training program? In the focus group interviews, these issues can be discussed between the participants, and they can respond to each other's experiences. The individual interviews are intended to allow reflection on own practice and the context it is part of. In relation to transfer, the interviews after the final modules will focus on what improvements, if any, the participants experience and how these improvements have influenced their ability to manage well-being and the psychological working environment in their wards. Furthermore, which factors they believe have contributed positively to implementation and transfer of the leadership training, as well as the issues that may pose a potential barrier and need to be addressed for the training to have the desired impact.

#### Participant observation

Participant observation during selected modules will be conducted by a researcher to experience first-hand how the interaction and exchange of knowledge and experiences take place between the participating managers and the HRD consultants. Participant observation will also provide information on how the managers experience and respond to the training during modules.

In addition to the above-described methods and to facilitate the following analysis of interview and observation data, various other issues relevant to the process evaluation are continuously recorded.. This includes the tools and exercises used in the training and information on how these are perceived by the managers. Documents are also collected ongoing, such as written materials developed in the intervention for participants or emails between the research team and the participating managers.

##### Analysis of qualitative data

Data from individual interviews, focus group interviews, and participant observations are used to examine the different CMO configurations related to whether the intervention was implemented as planned (e.g., whether recruitment proceeded as expected, whether planned activities were held as planned), which contextual factors influenced the intervention (e.g., conditions in the wards or other events that may have influenced the course of the intervention), whether participants' preconceptions and behaviours supported the intervention (e.g. whether their preconceptions of management, well-being and psychosocial working environment were compatible with the training's focus on self-care [59]. Other factors that have been identified that may have influenced the ability of workplaces and the managers to sustain any changes brought about by the intervention [51] will also be included in the analysis.

All interviews will be transcribed in full, and together with notes from participant observation, and other data (for example, notes from the participant observation in the group exercises), data are systematized and processed using the NVivo software [60]. An interpretive content analysis [61] will be conducted to explore how frontline managers experienced participating in the leadership training, to identify which factors influenced their participation, what their benefits were, and to elucidate which opportunities they had to transfer and what they gained from the leadership training into practice in their own wards and departments (see CMOs above). In order to do so, the material is coded in themes related to context, mechanisms and outcomes (deductive approach) but also coded according to how the leadership training is experienced (emerged) by the participants (inductive approach).

### Ethics and dissemination

#### Ethical approval

The study has received ethical approval from the Institutional Review Board at Aarhus University (2022–056).

#### Consent

For the participating managers and their employees, active informed consent was a prerequisite in order to participate in the study. The participating managers gave their informed consent, when they signed up for the study, while employees will provide their informed consent prior to completing the baseline questionnaires.

#### Confidentiality

To preserve confidentiality, the quantitative data is pseudo anonymized as soon as possible. All data are stored only on protected network drives with log-security, where data is only accessible to researchers involved in the project.

#### Dissemination of results

The data will be used to produce (1) scientific articles using the data, (2) dissemination articles and stakeholder dialogues, and (3) published trends, including a data report. Disseminated results will always be aggregated and it will never be possible to identify individuals in the results.

## Discussion

Action towards protecting and promoting the mental health of hospital employees and managers is essential to maintain a strong, well-functioning, healthcare system and to provide sustainable employment in the healthcare sector. In response to recent frameworks [7, 62, 63] there is increasing recognition that best practice in workplace mental health requires an integrated approach that prevents harm and promotes positive mental health [7]. There is also a greater understanding of the key role of managers in taking care of well-being in the workplace, but they often report a lack of knowledge, competencies, and values to handle their own and their employees’ health. This has prompted the implementation of training managers in facilitating a healthy psychosocial work environment to benefit well-being and mental health among staff and managers themselves. Systematic leadership training can be an important method for addressing these challenges. Given the strain on both managers and employees, we propose that effective leadership training should most likely target both manager self-care and staff-care. Self-care can be one way to increase managers’ attention and ability to handle their own health, and thereby in turn put them in a better situation to promote staff-care. To the best of our knowledge, this is the first research trial of its kind to be conducted in a Danish research setting. There are several methodological strengths to this study, including the randomized, waitlist, controlled design, the long follow-up period, and the collection of both questionnaire-based and register-based outcomes. Furthermore, the co-development of program design and content with HRD consultants was conducted, to ensure that the course material is meaningful and has a good fit to the specific managers of the partnering organization. This aligns with guidelines on developing and implementing organizational interventions [42]. An evaluation of a leadership training program in the healthcare sector is critical to promote mental health, but it is also a hard test of the training program itself, given the high demands and limited resources available in the sector. If the leadership training program is found to be effective in this setting, it will be an important step for Danish healthcare organizations in creating mentally healthy workplaces through the implementation of leadership training targeted specifically towards well-being and the psychosocial work environment. The value of the findings from this study have the potential to extend beyond the healthcare system and may influence other high-risk industries to address mental health in the workplace.

The thorough process evaluation employed in our study aims to provide a greater understanding of the barriers and facilitators of the contextual setting. One potential limitation to the study, however, may be the currently high level of staff turnover among hospital staff. The inability to follow up on employees and managers from baseline to follow-up due to staff turnovers may affect the likelihood of obtaining a robust understanding of the long-term effects at the secondary endpoint on the employee level. Another challenge may be the waitlist control design in which managers in the control group are asked to wait more than 12 months before their training starts. In order to maintain their interest in participating and to minimize the risk of participants in this group seeking leadership training elsewhere, the control group will be provided with activities and regular communication during the waiting period. Although it poses a conservative test of the intervention that control group managers are potentially seeking other forms of training, this can also reduce the possibilities of finding differences between the groups. This protocol has been written in accordance with the SPIRIT guidelines (see Appendix 1).

## Supplementary Information

**Additional file 1.**

## Data Availability

Not applicable.
